# Designs of Biomaterials and Microenvironments for Neuroengineering

**DOI:** 10.1155/2018/1021969

**Published:** 2018-12-09

**Authors:** Yanru Yang, Yuhua Zhang, Renjie Chai, Zhongze Gu

**Affiliations:** ^1^State Key Laboratory of Bioelectronics, Southeast University, Nanjing 210096, China; ^2^Key Laboratory for Developmental Genes and Human Disease, Ministry of Education, Institute of Life Sciences, Southeast University, Nanjing 210096, China; ^3^Co-Innovation Center of Neuroregeneration, Nantong University, Nantong 226001, China; ^4^Institute for Stem Cell and Regeneration, Chinese Academy of Sciences, Beijing 100101, China; ^5^Jiangsu Province High-Tech Key Laboratory for Bio-Medical Research, Southeast University, Nanjing 211189, China

## Abstract

Recent clinical research on neuroengineering is primarily focused on biocompatible materials, which can be used to provide electroactive and topological cues, regulate the microenvironment, and perform other functions. Novel biomaterials for neuroengineering have been received much attention in the field of research, including graphene, photonic crystals, and organ-on-a-chip. Graphene, which has the advantage of high mechanical strength and chemical stability with the unique electrochemical performance for electrical signal detection and transmission, has significant potential as a conductive scaffolding in the field of medicine. Photonic crystal materials, known as a novel concept in nerve substrates, have provided a new avenue for neuroengineering research because of their unique ordered structure and spectral attributes. The “organ-on-a-chip” systems have shown significant prospects for the developments of the solutions to nerve regeneration by mimicking the microenvironment of nerve tissue. This paper presents a review of current progress in the designs of biomaterials and microenvironments and provides case studies in developing nerve system stents upon these biomaterials. In addition, we compose a conductive patterned compounded biomaterial, which could mimic neuronal microenvironment for neuroengineering by concentrating the advantage of such biomaterials.

## 1. Introduction

Nerve lesions, which cause a great number of disabilities around the world, have brought a tremendous impact on patients' productivity and life quality. In general, nerve regeneration is the prime hindrance to limb reattachment in clinical practice. In previous studies, neuroengineering research for the peripheral nervous system (PNS) is primarily concentrated on alternatives to neurografts; however, work on spinal cord damage is primarily focused on creating a permissive environment for functional recovery [1]. During embryogenesis, neuron precursor cells (i.e., the neuroblasts) are divided and differentiated into the cellular components of the PNS and the central nervous system (CNS). They are driven towards specific cellular fates while migrating to predetermined destinations, and ultimately, these cells are developed into neurons and glial cells [2]. Nerve tissue engineering (NTE) is one of the most promising strategies for restoring CNS function in humans; in reality, the growth and distribution of cells within three-dimensional (3D) microporous scaffolds is of clinical significance for neuroengineering. Furthermore, NTE provides an attractive and promising platform for the competent management of PNS injury, by mechanically bridging the gap between severed nerves and also by inducing neuroregenerative mechanisms in a well-regulated environment that mimics the in vivo microenvironment of the specific nerve types that have been damaged so as to provide optimal clinical effectiveness [3]. For the existing explorations of different bioderived materials, they have provided several novel possibilities for the treatment and recovery of nerve injuries.

Nerve scaffolds consist of natural biological materials and synthetic materials. Thus far, many solutions have been introduced to derive the above two kinds of materials. For example, in [4], the authors derived the natural biological materials from autogenous nerves or other native tissues such as skeletal muscles or blood vessels as well as polyester materials, i.e., polyhydroxyalkanoate. Another idea for natural biomaterials is to develop tissue-engineered nerve scaffolds by reconstituting nerve cell-derived extracellular matrix (ECM) using natural biomaterials and then develop a protocol to prepare and characterize cultured Schwann cell-derived ECM [5]. For the above protocol, silk fibroin fibers and a chitosan conduit are prepared, seeded with Schwann cells for deposition of ECM, and suffered from decellularization. However, this was confirmed to assembly into a Schwann cell-supported, chitosan/silk fibroin-mediated, and ECM-coated scaffold which was used to bridge a 10 mm gap in the sciatic nerve of rat. On the other hand, the synthetic materials used in nerve scaffolds mainly include decalcified bone tubes, nylon fiber tubes, and polyurethane. Yang et al. developed microporous polymeric nanofibrous scaffolds through biodegradable poly(l-lactic acid) (PLLA) for a two-dimensional (2D) nerve stem cell (NSC) culture. The production of PLLA scaffolds is carried out by a liquid-liquid phase separation strategy. This indicates that the physicochemical features of the scaffolds have been fully characterized, by scanning electron microscopy and differential scanning calorimetry [6].

The combination of materials and tissue engineering is a mature research field, and numerous materials have been applied in clinical therapies. In recent years, several novel materials have been exploited, which is mainly due to the fact that their excellent chemical and physical features have been applied to the field of neuroengineering. In particular, a clinical study has been performed to determine the feasibility and safety of the collagen scaffold NeuroRegen, and it is found that patients demonstrated improved autonomic nerve function, and meanwhile, there is a recovery of motor- and sensory-evoked potentials from the soma [7]. The combination of biomaterials and neuroengineering has been widely researched all over the world; however, it is necessary to develop more novel materials to provide more choices for clinical therapy.

Graphene consists of a single layer of carbon atoms, which have a high mechanical strength and chemical stability with unique electrochemical properties for electrical signal detection and transmission. This is important, due to the fact that, the promote diagnosis and nerve disease treatment mainly rely on the stimulation and recording of nerve impulses. A great number of biomaterials have been adopted as nerve scaffolds, and organ-on-chip devices provide novel in vitro microenvironments, which make progressed in the point where they are able to be used in the development and regeneration of nerve tissue. In reality, the latest progress in microtechnology allows more realistic mimicking of the naturally occurring microenvironment, where the behaviors and physiology of neurons and NSCs in response to the physical environment are more realistic. Neural interface biomaterials have become a topic of great interest; meanwhile, photonic materials are an emerging area in the production of stents in NTE. Ordered porous materials like photonic crystals provide a surface effect for studying the behavior of NSCs.

This literature review covers recent studies on the three kinds of the above bioderived materials and their neuroengineering applications. Exploring the application of the compounded biomaterials in the field of neural interface materials could be serviceable in fabricating multifunctional neuron scaffold, which can be used not only for in vitro studies but also for therapeutic purposes. In addition, we combine the merit of such biomaterials to develop a compound design which has the advantage to further improve nerve cell growth.

## 2. The Growth of Nerve Tissue Guided with Graphene

Neurons are electrically active cells, which function is exceedingly closely related to electrical activity. Through depolarizing the excitable cell membranes, electrical stimulation can initiate a functional response in neurons. In theory, depolarization can be achieved by the ionic flow between two or more electrodes; meanwhile, at least one of the electrodes is close to the target tissue. In general, there are two categories of electrodes that have been used in neural stimulation in neuroengineering research. Microelectrodes show low-charge/density thresholds and high-charge/phase thresholds, and they are, respectively, fixed on the target organ surface and possess a geometric surface area (GSA) greater over approximately 100,000 *μ*m^2^ [8, 9]. Researchers have confirmed that electrical charges are able to enhance nerve regeneration by altering protein adsorption during neuron interactions with electroconductive materials [10, 11]. Scaffolds designed for neuroengineering can simulate the electrical properties of neurons. The results show that the growth on the conductive substrate can enhance neurite growth under electrical stimulation [12–14].

In general, graphene is the strongest and thinnest known material, which has been received great attention since being separated from graphite by Novoselov and Geim in 2004 [15, 16]. Graphene, also known as a single-crystal graphite, is a 2D crystal which consists of a single layer of carbon atoms. It is worth mentioning that the large specific surface area, excellent thermal, mechanical, and optical properties, as well as its outstanding electrical conductivity, make graphene an obvious choice for guiding the growth of nerve tissue. The work of Fabbro et al. has shown that untreated graphene can be connected to neurons, and the graphene has the ability to maintain the integrity of the active cells. This work is the first time to demonstrate that graphene can control the first step in creating deep brain implants, and graphene electrodes have great promise for implantation in the brain, which is able to restore functional loss after amputation, to reverse paralysis, and to provide relief for patients with movement disorders such as Parkinson's disease [17]. The existing researches on neuroengineering are focused on studying the impacts of the graphene sheets on the complex relationship between neuron signal transmission mechanisms, and many works have shown that the diverse physical properties of graphene can affect the directional growth of neuronal axons, which can be used to promote the growth and activity of NSCs.

### 2.1. Graphene as a Two-Dimensional Substrate for Neurons

The combination of outstanding thermal stability, biocompatibility, mechanical strength, and high electrical conductivity makes 2D graphene promising for a host of bioengineering applications [18, 19]. Graphene oxide (GO) is superior to graphene for the preparation of homogeneous aqueous suspensions in the existence of oxygen-encompassing hydrophilic groups, which decrease the reversible agglomeration of the graphene sheets. There has also been work showing that ginseng-reduced graphene oxide (rGO) sheets increase the differentiation efficiency of NSCs towards nerve cells. In one experiment, hydrophobic hydrazine-rGO films exhibited no toxicity against human neural stem cells (hNSCs), and the hydrophilic GO and ginseng-rGO films (which are more biocompatible films) showed proliferation of the hNSCs after three days. In addition, the hydrazine-rGO and peculiar ginseng-rGO films exhibited greater differentiation of hNSCs into neurons (rather than into glial cells) than the GO film after three weeks. The higher capability for electron transfer with rGO films bring about the promoted differentiation on such films [20]. However, when compared to graphene and other 2D or quasi-two-dimensional nanostructures that manifest superior flexibility and conductivity, the rGO derivative exhibits more worse conductivity [21]. The 2D graphene used in the work with nerve cells is mainly produced by chemical vapor deposition [22, 23]. Zhang et al. measured the cytotoxicity of graphene layers in neural phaeochromocytoma-derived PC12 cells and found that graphene induced strong metabolic activity at low concentrations, while the cell apoptosis marker (caspase-3) was activated in large numbers when PC12 cells were exposed to graphene at high concentration of 10 g/m [24]. Li's group mainly researches on the effects of 2D graphene film on the development of hippocampal neuron cells, and they have demonstrated that graphene not only has favorable biocompatibility with neurons but also plays a significant role in promoting neurite sprouting and outgrowth of mice hippocampal cells [25]. This work manifests the prospect of graphene as a biomaterial for neural interfacing and offers insight into the future bioengineering applications of graphene.

### 2.2. Graphene as a Three-Dimensional Nerve Scaffold Material

In vitro experiments on cell behaviors in the presence of graphene usually involve 2D graphene films, which lead to discrepancies between the 3D in vivo environment and the artificial 2D environment. Compared with the 2D scaffolds, 3D scaffolds are more accurate to mimic the chemical, physical, and biological properties of the in vivo environments [26–28]. Due to their interconnected porous structure and larger specific surface area, the 3D micropores of graphene make it an excellent scaffold material for regenerative medicine and tissue engineering and for providing a biomaterial interaction platform in living organisms during in vivo experiments [29–31]. The existing literatures have demonstrated that the topographical cues, including the sizes and patterns of biomaterials, have great influences on the NSC behavior. In reality, these observations provide a better understanding of the different roles that mechanical transduction plays in stem cell fate, especially in terms of directional differentiation, and how these dynamic cues can be used to advance the field of stem cell therapy [32].

To develop practical applications for graphene, significant effort has gone into assembling 2D graphene sheets into 3D macroscopic structures that can serve as nerve scaffolds. The characteristics of 3D graphene are closely related to the size of such structures. Therefore, carefully controlling the size of the 3D preparation allows one to regulate the topographical cues of graphene, which can be used to meet different application requirements, and provides the opportunity to better understand the mechanism behind graphene effects in different applications. Many researchers have shown that 3D graphene stents cannot only promote the propagation of NSCs but also induce the selective differentiation of NSCs into functional neurons to a certain degree. For instance, porous three-dimensional graphene foam (3D-GF), which acts as a novel scaffold for NSCs, cannot only maintain NSC growth but also support the cells in an active propagation state through upregulation of Ki67 expression, when compared with 2D graphene films. It has also been shown that 3D-GFs can accelerate the differentiation of NSCs into astrocytes and neurons; meanwhile, the electrical coupling of 3D-GFs with differentiated NSCs demonstrated the effective electrical stimulation of these cells [33].

In Tang's [34] and Song's [35] research group, a novel interconnected micropore scaffold 3D-GF is introduced for NSCs in vitro, which can be used to carry out a more in-depth study of the effects of 3D graphene on the cell. Their study found that microglial cells can grow very well on 3D graphene, and the pattern of graphene/cell interactions has an influence on the pro- and anti-inflammatory responses of microglia cells, which are cultured on graphene film or 3D-GF. Graphene showed a remarkable ability to rescue LPS-induced neuroinflammation, most likely through the restriction of microglial morphological transformation by the topographical cues of the 3D-GF surface. It is worth mentioning that hydrogel-doped graphene possesses fantabulous flexibility, which has received great attention for improving the regeneration of the PNS. Furthermore, the wettability, swelling ratio, morphology, mechanical properties, composition, and degradation behavior of graphene oxide/polyacrylamide (GO/PAM) hydrogels have been well characterized, and GO/PAM hydrogels have behaved a positive impact on Schwann cell adhesion and propagation [30].

### 2.3. Graphene with Other Applications in Neuroengineering

In previous studies, a novel method is introduced to inhibit synapses by fabricating nanometer-scale GO fragments. This solution mainly affects cell activity rather than inhibiting neuron signaling, which has been widely used in the treatment of neurological diseases [18]. Given the superior properties of 3D graphene structures, the synapses will be applied in neuroengineering and NSC transplantation treatment and other fields.

Graphene functions as an improved artificial graft can be used to support nerve repair and regeneration. The unique physical properties regulate cellular growth behavior and improve cell activity, function, and development. In the future, the primary goal of biomedical engineering will be to address the potential applications of using graphene as a support biomaterial in cell culture. For example, the conductive properties of graphene will allow us to apply directional electric current on living tissues. In summary, graphene addresses quite a few challenging clinical applications of bioengineering and has great prospects in neuroengineering.

## 3. The Application of Photonic Crystals in Neuroengineering

In 1987, Yablonovitch and John put forward the new concept of “photonic crystals” which expounds upon the effect that periodic dielectric structures have on the way that light propagates through certain crystalline materials. Photonic crystals consist of ordered arrays of two or more materials with different dielectric constants (refractive indexes). The materials form periodic patterns of dielectric constants, which generate a range of “forbidden” frequencies referred to as the photonic bandgap, and photons with energies in the bandgap cannot propagate through the material [36]. Although there are examples of photonic crystals in nature, such as opal, feathers, and butterfly wings, the vast majority of photonic crystals are of artificial design. A number of artificial fabrication techniques are currently available to achieve responsive photonic crystal patterning [37–40], and the application of photonic crystal materials is an emerging research field for novel nerve scaffolds, which can be used as stents in neuroengineering.

### 3.1. The Guidance of Nerve Cells by Ordered Structure

Recently, the topological cues provided by biological scaffolds have been suggested to regulate cell behavior and stem cell fate [41–44]. These large structures can be measured in micrometers, and much work has been gone into determining their organization, assembly, molecular composition, and function [45]. The research results have shown that substrates patterned with grooves or ridges can regulate cell adhesion and orientation [46]. In addition, it has been shown that the morphology and alignment of cells can be modified by culturing them on stretched polymer inverse opal films [47]. In support of the applicability of such technologies, studies have shown that there is a substantial connection between NSC behavior and the nanotopography of the materials upon which they are growing [48, 49].

The development of photonic crystal microstructures has been a primary focus of research into tissue regeneration over the past thirty years, and these materials have found applications in a multitude of tissue engineering applications, such as controlling the spatial arrangement of cells, guiding cell behavior, and differentiating stem cells. To be specific [50], proposed the application of uncomplicated stretched inverse opal structures for guiding the formation of cell orientation gradients, and it was shown that tendon fibroblasts growing on such structures formed elongation gradients that matched the topographical cues of the ordered substrate [50]. Thus far, there have been reports of applying photonic crystal structures in neuroengineering, as shown in [51–53].

Nerve cell synapses can be easily guided by mechanical force in vivo, and the “random-to-aligned” cell gradients generated by such forces reproduce the part of the neuron that is inserted into connecting tissues and has significant potential for applications in neuroengineering. Photonic crystal materials like ordered microporous silicon are promising electrode materials in the nerve repair setting, which is mainly due to their advantage of biologically inert with excellent biocompatibility. Porous silicon has a large surface area, adheres firmly to tissues, and does not induce inflammatory response; all of which suggest that it would make a good biomaterial for use in implantable electronic nerve devices [51]. Wang et al. have developed a novel approach to create microporous tubular scaffolds from chitosan, which have mechanical properties and controllable inner structures, and therefore, they are useful for neuroengineering. The material has highly porous inner matrices with a large network of interconnected pores and axially oriented microchannels. Experiments in living donor tissue showed that these scaffolds exhibit mechanical strength, swelling, porosity, and biodegradability, which mimic the physical and chemical microenvironment in living organisms, and therefore will be of great potential for applications in neuroengineering. Characterization of in vitro cell cultures on these chitosan scaffolds showed that differentiated Neuro-2*α* cells grew along with the oriented microchannels, and the interrelated pores in the scaffold's interior were beneficial for both nutrient diffusion and cell ingrowth [52]. The adoption of patterned biomimetic materials can guide the growth and arrangement of cells [54], and ordered porous materials provide a surface effect for the study of nerve cells and the behavior and effect of NSCs.

### 3.2. Monitoring Nerve Cells on Ordered Porous Material

The photonic bandgap of periodic dielectric structures is the fundamental property of photonic crystals. The emergence of the photonic bandgap relies on the structure of the crystals, the ratio of the dielectric constants of the materials making up the crystal, and the geometric configuration of the crystal. In general, if the difference in the dielectric constant between the two kinds of material in the photonic crystal is obvious enough, then Bragg scattering will occur at the medium interface, and the dielectric constant ratio will become greater. In addition, the stronger the incident light is scattered, the greater the possibility to generate a photonic bandgap [55, 56]. The characteristic reflection peaks of the crystals is determined by the structural periodicity, herewith, the ordered porous crystals exhibit a perfect inertness because they can avoid chemical instability such as bleaching, quenching, or fading [57].

In addition to the significant physical features with which periodic dielectric structures can guide the growth of nerve cells, photonic crystals can be used in a number of applications, which make use of the photonic bandgap. For example, their long-range ordered structures provide a stable code that can direct the growth of nerve cells according to changes in the refractive index. Huang et al. presented that lithographically patterned microporous silicon photonic crystals, which are functionalized with different bioactive peptide-doped surfaces, could be used as a spatial guidance for NSC differentiation and that NSCs can be spatially specified to suffer astrogenesis or neurogenesis as a multifunction of peptide identity as well as surface properties [58]. In addition, these crystals have found applications in the field of biomedical optics, and it has been shown that adsorbing proteins to the surface of a photonic crystal changes the refractive index which can be used to detect neurotransmitters and neural markers. To be specific, acetylcholinesterase-based organophosphate nerve agent-sensing photonic crystals have been widely studied in neuroengineering. These photonic crystals consist of polymerized crystalline colloidal arrays that can detect the organophosphorous compound parathion at ultratrace concentrations in aqueous solutions, and the sensor will cause a red shift in the wavelength of the diffracted light if it detects the nerve agent [59–62].

## 4. Organ-On-a-Chip as a Microsystem for Nerve Tissue

The engineering of cellular environments has been shown to be crucial for improving the in vitro viability and in vivo-like function of cells and tissues, which is due to the fact that such environments are more accurate to mimic the situation in living organism [63]. Organs-on-chip platforms, including microfluidic, microengineering technologies, and essential bionic principles to faithfully describe the significant aspect of tissues in living organism, consist of critical spatiotemporal, microarchitecture cell-cell communications, and extracellular environments [64]. The improvement of organ-on-a-chip devices has yielded practical applications in drug screening and clinical research. The in vitro organ-based experiments with this new technology carry on the historical tradition of medical techniques that have sought to reconstitute damaged organs or tissues, and this novel technology is especially relevant to the research on the nervous system. Lundborg has proposed that the implantation of microfluidic chips in the nervous system might offer a novel interface between biology and technology, the concomitant development of gene engineering might provide novel possibilities for the manipulation of nerve regeneration and degeneration [65]. Organ-on-a-chip technology overcomes many of the challenges traditionally associated with clinical studies of neurological disease, especially when it comes to the complexity of neurological phenomena. The combination of neural engineering and chip research at present is mainly focused on axonal growth, the blood-brain barrier, neurospheres, and 3D or layered neural tissues [66–71].

### 4.1. Axonal Growth on a Chip

To successfully regenerate nerve tissue, axonal outgrowth from the proximal stumps requires growth without interference from the surrounding microenvironment, while it requires the formation of new connections with distal stumps. To address the above issue, Bryan et al. proposed a novel strategy to improve axonal sprouting in a guided way through a spatial neuron guidance channel [72]. Studies have shown that microchannels or microgrooves ranging in size from a few dozen nanometers up to 10 microns in width can induce directional axon growth [73] and meanwhile have a promoting effect on the formation and development of axons [74, 75]. In addition, bioresorbable guide channels, which are made of poly(lactic-co-glycolic) acid, have been shown to greatly affect the glial growth factors and Schwann cells during peripheral nerve regeneration [72]. Novel protocols for establishing CNS models on microplatforms have allowed axons to be visualized and quantified [76], and Hadlock et al. found that a polymer foam conduit comprised of some microchannels aligned longitudinally, which the diameters range from 60 to 550 microns and cultured with Schwann cells, promoted the regeneration of peripheral nerve. These conduits offer a microenvironment that is permissive to axonal regeneration [77].

Kim et al. created the neuron chellop through a surface-printed microdot array to control axon branch formation and showed that the majority of collateral axon branches stemmed from axonal regions on a dot and terminated on neighboring microdots. In that study, the results showed that the length of branches increased as the spacing between dots increased [8]. This approach was also used to identify connectability defects in nerve cells from mouse model of 22q11.2 deletion syndrome/DiGeorge syndrome, by comparing the applications of channel guides to wild-type preparations. The results of that experiment demonstrated the reliability and sensitivity of the on-chip connectability approach and validated that tackling measures for quick assessment of neuron connectability defects in neuropsychiatric disease modeling [78]. The application of microchips in neuroengineering is appealing on account of their ability of maintaining the cellular environment in both a spatial and temporal manner [79]. Shin et al. designed a compartmental microfluidic device as a cell culture chamber and found that axons traversed the channels in microchips, which could be separated from the somata, thus forming an arrangement comparable to dissociated primary neurons [80]. The axons are the functional units that connect neurons to each other, and hence, the existing technologies such as those described here need to be further developed to provide the accurate guidance of axonal growth, which is needed for neuroengineering applications.

### 4.2. Microfluidic BBBs-On-a-Chip

The blood-brain barrier (BBB) is derived from specialized endothelial cells, which isolates the blood from the brain tissue. Specifically, the BBB hinders the access of many exogenous compounds to the CNS selectively [81]. It is worth mentioning that the BBB is basically consists of three kinds of cells, where endothelial cells lined along astrocytes and pericytes. The membrane forms large numbers of tight junctions among endothelial cells. Therefore, the compound permeability can be directly controlled by maintaining high levels of transendothelial electrical resistance. The BBB protects the brain from noxious compounds in the blood and offers a homeostatic environment for optimal neuronal function. Limited BBB permeability leads to low efficiency in the clinical drug treatment of CNS pathologies, and examining BBB dysfunction and function is crucial for biomedical studies and drug development [82].

The BBB-on-a-chip is a microfluidic platform, which can be used to mechanically and biochemically modulate BBB function [83]. This technology enables the real-time monitoring of neurons in a designed physiological niche, for example, through the use of small chambers and fluid guides as well as the attachment of sensors. Instances of BBBs-on-a-chip in the literature have demonstrated the feasibility of providing more accurate environments for research on organ-level functions [84–86]. These modular microenvironments recapitulate the roles of the “neurovascular unit” through a vertical stack of poly(dimethylsiloxane) neural parenchymal chambers, which are mainly separated from a vascular channel made of a porous polycarbonate membrane. Such microsystems will likely prove useful in studying neurodegenerative disorders and in toxicology and neuroinfectious disease studies as a screening tool [87].

Microfluidic devices are gaining ground as novel automated microsystems for neuron culture and real-time monitoring, and the BBB-on-a-chip model provides an in vitro environment to mimic the natural forms and functions of the BBB and might be of great benefit in developing methods for nerve disease and new clinical treatments [88]. For instance, recreating the BBB structure and physiology on a chip—which is a neurovascular microfluidic bioreactor incorporating both a brain chamber and a vascular chamber separated by a microporous membrane—allows for adequate cell aggregation to support real-time monitoring and systematic analysis [89]. In addition, organs-on-a-chip have opened up a novel avenue for researching the characteristics of neurons derived from Alzheimer's disease brains. A microfluidic chip based on 3D neuroaxonal spheroids is more accurate to imitate the brains in living organisms by supplying a constant quantity of fluid, which is similar to what is seen in the interstitial space of the brain. Furthermore, researchers have used such chips to study the influence of flow on neural networks, neurospheroid size, and nerve stem cell differentiation [90]. Takeda et al. designed a three-chambered microfluidic platform for modeling double-layered neurons to examine the ingestion and proliferation in response to changes in tau values, which occur in the interstitial fluid in the brains of tau transgenic mice and in the cortices of human Alzheimer's disease patients [91]. In summary, 3D microfluidic BBBs-on-a-chip with controllable size and shape are a potential in vitro model for studying nerve tissue disease.

## 5. Conclusions and Discussions

The unique morphology of nerves and neurons—with their distinct functional units—makes nerve repair and regeneration particularly challenging. The field of clinical medical materials has progressed significance in the past several years and has given rise to the design and synthesis of functional biomaterials for the therapeutic and diagnostic applications. The present challenges and the future goals for such proof-of-concept research need to be emphasized as well. Electrical conductivity is the primary predictor of neural signal quality in nerve repair and regeneration, and conductive materials will be useful for a great number of applications. It is worth mentioning that the communication between neurons and their downstream target cells takes place through the specificity of synapses, and information is transmitted between neuronal circuit elements by electrical or chemical signals. Neural signals based on electric conduction are predicted to have a strong influence on nerve reparation and regeneration, and neural components made of conductive material are expected to have wide clinical potential.

The development of graphene technology is a field at the frontier of biomedical research, and one of the primary focuses of such research is to find ways to manipulate the properties of graphene materials so as to modulate neuronal synapses and neuronal excitability. The directional morphology of cell culture scaffolds can promote various nerve cell behaviors, and thus, another promising technology is the stimulation of nerve cell growth by topological cues and electrical signals through the use of photonic crystals. These crystals are relatively easy to make. Specifically, they are highly conductive and controllable. There is still a significant room for the optimization of nerve conduits, because a number of the parameters which affect the clinical effects as well as the underlying mechanisms of their use are still not well understood [3]. Accordingly, there has only been limited exploitations of photonic crystal devices in neuroengineering. Microfluidic-based devices have emerged as new cell culture platforms for neurobiology research. This is due to their excellent spatial and temporal control capacities, easy assembly, reproducibility, flexibility, and amenability in imaging and biochemical analyses as well as their high-throughput potentials, which are likely to play an increasingly important role in establishing physiologically relevant culture/tissue models [92].

**Figure 1:**
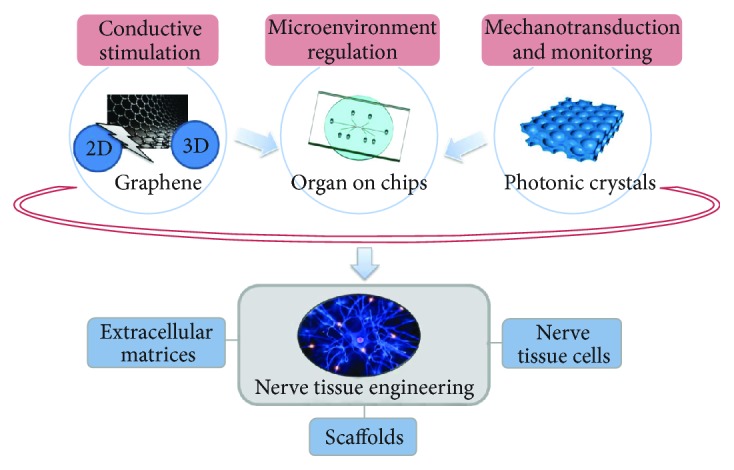
Design of biomaterial and microenvironment by graphene, photonic crystals, and organ-on-a-chip for NTE.

The evolution of biomimetic ECMs is of great significance in neuron tissue reparation and regeneration. The existing research has shown that electrostimulation of neurons in the absence of topological characteristics can guide axonal extension. During nerve repair and regeneration, the growth behavior of nerve cells is regulated by the directional morphology of a scaffold, the mechanical stretching of the scaffold, and the electronic signals in the scaffold. However, the existing studies do not consider the above factors together to regulate cell growth behavior. A technical problem for neuroengineering is how to develop a strategy that can combine the advantages of all these factors to further improve nerve cell growth (Figure 1). Accordingly, it is necessary to address these issues as a way of identifying more efficient and cost-effective therapies in future research. Such hybrid biomaterials will find use with myocardial and other tissues such as muscle or bone and will be useful for the fabrication of tissues and cell constructs by providing conductive media, topographical cues, and biomimetic microenvironments.
